# Understanding the dynamics of keratin weakening and hydrolysis by proteases

**DOI:** 10.1371/journal.pone.0202608

**Published:** 2018-08-16

**Authors:** Laura Navone, Robert Speight

**Affiliations:** Science and Engineering Faculty, Queensland University of Technology, Brisbane, Queensland, Australia; University of Tennessee, UNITED STATES

## Abstract

Keratin is the structural protein in hair, nails, feathers and horns. Keratin is recalcitrant, highly disulfide bonded and is generally inaccessible to common proteases. Only certain types of proteases, called keratinases, are able to cleave the peptide bonds within the keratin structure. Due to this outstanding activity, keratinases have potential application in industries such as livestock, cosmetics and pharmaceuticals. Yet, the process of enzymatic keratin degradation is poorly understood, affecting the development of industrial enzyme formulations that may require full or only partial modification or weakening. Here we investigate the dynamics of keratin weakening and hydrolysis, showing that the decrease in hair mechanical strength is associated with cuticle removal and damage to the cortex and complete breakdown is dependent on reducing agents. Proteases with keratinolytic activity were selected and applied to hair with degradation examined by mechanical, biochemical and microscopic techniques. The extent of keratin degradation was highly enhanced by the presence of reducing agents, principally sodium thioglycolate, exceeding 90% degradation within 16 h of enzymatic treatment. Application was extended to feathers showing that the findings are relevant to improving the use of keratinases in a variety of industries. Overall, the outcomes provide valuable insights into the keratin degradation process by enzymes for the optimization of cosmetic and pharmaceutical products and for livestock waste recycling among other important applications.

## Introduction

Keratin is a stable, insoluble and fibrous structural protein found in epithelial tissues (soft keratin) and protective tissues such as hair, nails, wool, feathers and horns (hard keratins) [1]. Based on the secondary structure, keratins can be classified into α-keratin or β-keratin [2]. β-Keratin is rich in β-pleated-sheets forming supramolecular fibril bundles [3] and α-keratin consists of α-helical-coiled coils self-assembled into intermediate filaments [2]. The strength and robustness of keratin is derived from these tightly packed α-helical and β-sheet configurations. Keratins contain a high degree of disulfide bonding, which confers rigidity and chemical resistance. Keratinaceous materials possess different amounts of α- and β- keratins [4]. For example, hair is mainly composed of α-keratin, while feathers are mainly formed of β-keratin [5, 6].

Keratinases are proteolytic enzymes capable of catalysing the hydrolysis of highly stable keratin proteins that compose hair and feathers, and other keratinous materials. Common proteases like pepsin and papain are not capable of degrading keratin. It is generally recognised that the keratinolytic process involves two steps, sulfitolysis and proteolysis [4, 7, 8]. During sulfitolysis, cleavage of disulfide bonds changes the conformation of keratins and more sites for keratinase action are exposed [9]. Since keratinases do not possess disulfide-reducing potential, reduction of disulfide bonds needs to be accomplished by either sulfide reductases [7, 10] or by secretion of reducing agents like sulfite [8, 11]. For example, dermatophytes growing on keratin-rich structures like nails, hair and skin, are known to secrete sulfite to facilitate keratin degradation together with proteases [8]. The release of sulfite supports the proteolytic action of secreted keratinases enhancing access to the substrate and resulting in the generation of oligopeptides and amino acids for growth of the dermatophyte [8].

Keratinases have been studied from various groups of bacteria and fungi [12–18]. The first keratinase purified and characterized was KerA from a *Bacillus licheniformis* strain that was shown to be a subtilisin-like protease belonging to the serine protease S8 family [12]. Other keratinases from *Bacillus* sp. and *Streptomyces* sp., also belong to the S8 family [19]. Keratinases belonging to the metalloprotease M14 and M4 families have also been identified from diverse groups of bacteria and fungi [13, 15, 16, 20]. Even though many keratinases have been recognized, a small number have reached commercial production to date. The limited knowledge of the process of enzymatic keratin decomposition has in part impaired additional development of keratinase products. Some personal care products for cosmetic skin whitening, removal of calluses, treatment of acne, hair care and anti-dandruff shampoo are available that contain keratinases [21–24]. In the pharmaceutical industry, keratinases have raised attention due to their prion decontamination capability [25]. Keratinases could be used for the cleaning of keratinous wastes generated from feathers, hair, horns, hooves and nails by meat and leather processing industries. Keratinous waste is conventionally hydrolysed by chemical treatments that are inefficient and polluting [25, 26]. Keratinases could replace chemical treatments, efficiently degrading waste and generating valuable products as a result. Keratin hydrolysate has a high nitrogen content and is rich in hydrophobic amino acids, making it a useful product for applications such as in animal feed, bio-fertilisers and cosmetics [4, 27].

Most research on microbial keratin degradation has been performed using whole cell culture broth to test for decomposition capabilities of microorganisms and not focused on the study of the individual components involved in degradation. To develop an effective keratin degradation product, isolated components have to first be characterized to optimize system design. Further understanding keratinase performance towards specific substrates would facilitate the generation of new formulations targeted to particular applications.

In this work, we studied enzymatic degradation of hair and feathers using commercially available proteases and investigated the effects of two common reducing agents, sodium sulfite and sodium thioglycolate. While sodium sulfite is a known abiotic component of microbial keratin degradation [8], thioglycolate salts have been extensively used in hair cosmetic products for waving or straightening [28–31]. We show that removal of the hair protective cuticle and damage to the cortex affects mechanical strength and that the presence of a reducing agent is crucial for complete keratin degradation by keratinases. Time series analysis of enzymatic treated hair and feathers using scanning electron microscopy revealed well-defined stages of keratin degradation, showing that the extent of keratin weakening and hydrolysis can be controlled and optimized for specific applications.

## Materials and methods

### Ethics statement

Chicken feathers were obtained from a non-commercial source. Hair was obtained from a medium-sized cattle farm in the state of Queensland. No approval from the University Animal Ethics Committee at Queensland University of Technology was required for this study.

### Protease activity determination

Protease activity was determined using azocasein (Sigma Aldrich). Fifty μL of diluted enzyme solution was added to 50 μL of 2% azocasein in 100 mM Tris-HCl, pH 8 and incubated at 37°C for 30 min. Non-digested azocasein was precipitated by addition of 100 μL of 10% trichloroacetic acid (TCA) to each incubation, kept on ice for 10–15 min and centrifuged at 4,500 × g for 10 min at room temperature. 100 μL of supernatant was transferred to a 96-well microtitre plate containing 200 μL of 1 M NaOH and the absorbance was measured at 440 nm [17]. Determinations for each enzyme dilution were performed in triplicate. Negative controls were prepared by precipitating the azocasein substrate with TCA and followed by addition of the enzyme dilution without incubation. Increased absorbance indicates the presence of proteolytic activity. One azocasein unit (CU) was defined as an increase of 0.1 absorption units after incubation for 30 min at 37°C. Total protein was determined by Bradford assay [32]. For keratin absorption experiments enzymes were incubated with hair for 1 h at 37°C. Tubes were centrifuged and supernatant assayed for protease activity with the azocasein assay. Remaining activity was calculated as a percentage of protease activity without hair incubation.

### Keratinase activity determination

Keratinolytic activity was determined using keratin azure (Sigma Aldrich) following the manufacturer’s instructions with some modifications. Briefly, 100 μL of enzyme dilution was added to 0.01 g of keratin azure in 2.4 mL of 100 mM Tris-HCl buffer pH 8 or pH 10. Samples were incubated at 37°C for 1 h at 200 rpm. After incubation, samples were centrifuged at 4500 × g for 10 min and the absorbance of the clarified supernatants was determined at 595 nm. Determinations for each enzyme dilution were performed in triplicate. For the negative control, 0.01 g of keratin azure in 2.5 mL of reaction buffer was incubated at 37°C for 1 h with shaking at 200 rpm and the absorbance was measured at 595 nm. One keratin unit (KU) was defined as an increase of 0.1 in absorbance at 595 nm after incubation for 30 min under the experimental conditions described. For keratinolytic activity determination in presence of reducing agents, the keratin azure assay was performed as indicated with the addition of 1 or 2% of sodium sulfite or 1, 2 or 5% of sodium thioglycolate. Total protein was determined by Bradford assay [32].

### Hair breakage force studies

Hair samples of 0.01 g from cow hides (*Bos taurus*) were treated with 0.2, 1 or 2 KU/mL of Multifect PR 6L or Cibenza DP100 in 5 mL of 100 mM Tris-HCl buffer pH 8, and 0.02, 0.1 and 0.2 KU/mL of Ronozyme ProAct in 5 mL of 100 mM Tris-HCl buffer pH 10, for 16 h at 37°C at 200 rpm. Control hair samples were incubated in reaction buffer without enzyme for 16 h at 37°C at 200 rpm. A MTS Microforce Tryton 250 force meter was used for axial force testing studies. Dry hair samples of 4 cm length for each condition were loaded into two horizontally aligned clamps and displaced 20 mm over 40 s (0.5 mm/s strain rate) for breakage testing at room temperature and ambient relative humidity (~50%). Hair force to break was defined as the load in Newtons required for hair rupture and the tests were repeated ten times for each condition. Statistical analysis was performed using a one tailed- distribution t-test (p ≤ 0.01).

### Determination of soluble peptides and percentage degradation after hair or feather treatment

Hair samples of 0.01 g from cattle hides or chicken feathers samples of 0.01 g, were treated with Cibenza DP100 or Ronozyme ProAct in 5 mL of 100 mM Tris-HCl buffer pH 8 or pH 10, respectively, for 16 h at 37°C in 50 mL tubes with agitation at 200 rpm. Where indicated, 1% sodium sulfite or 2% sodium thioglycolate was added. Control hair and feather samples were incubated in reaction buffer without enzyme for 16 h at 37°C at 200 rpm. Reducing agent was added where indicated. Soluble peptides were quantified by Bradford assay after 2, 6, 10 and 16 h of incubation [32]. Percentage of degradation was determined by weight loss. Remaining hair and feather samples after treatment were centrifuged, supernatant removed, and left to completely dry for several days. Final weight was measured and percentage degradation calculated by subtracting initial weight.

### Scanning electron microscopy

Hair and feather samples treated with Cibenza DP100 and Ronozyme ProAct for 2, 6, 10 and 16 h at 37°C, with or without reducing agent (1% sodium sulfite or 2% sodium thioglycolate), were centrifuged and air dried for several days, fixed in a sample holder stub and gold coated using a Leica EM SCD005 Gold Coater (to ~ 10 nm). Secondary electron images were obtained with Zeiss ∑igma Field Emission Scanning Electron Microscope. Images were obtained under vacuum using 2 kV accelerating voltage.

## Results

### Enzymatic activity of commercial proteases

Commercial proteases for this study were obtained from different companies, Cibenza DP100 from Novus, Ronozyme ProAct from DSM-Novozymes, Multifect PR 6L from Dupont, Alcalase 2.4 LT, Neutrase 0.8 BrewQ and Flavourzyme from Novozymes (Table 1). These products are commercialized as feed additives (Cibenza DP100 and Ronozyme ProAct) or as proteases for industrial food manufacturing processes (Multifect PR 6L, Alcalase 2.4 LT, Neutrase 0.8 BrewQ and Flavourzyme). Cibenza DP100 is the only product known to be marketed as a keratinase [33].

**Table 1:** Commercial enzymes used in this study.

Product Name	Stated enzyme type(s)	Enzyme source organism	Company
**Cibenza DP100**	Protease/Keratinase	*B*. *licheniformis* PWD-1	Novus
**Alcalase 2.4 L FG**	Protease (Subtilisin)	*B*. *licheniformis*	Novozymes
**Neutrase 0.8 L BrewQ**	Protease	*B*. *amyloliquefaciens*	Novozymes
**Flavourzyme**	Aminopeptidase	*Aspergillus oryzae*	Novozymes
**Multifect PR 6L**	Protease (Subtilisin)	*B*. *licheniformis*	Dupont
**Ronozyme ProAct**	Protease	*Nocardiopsis prasina*	DSM/Novozymes

The substrate specificity of each enzyme towards casein and keratin was determined by azocasein or keratin azure assays respectively (Table 2). The commercial products Cibenza DP100, Ronozyme ProAct and Multifect PR 6L all showed keratinolytic activity, while Alcalase 2.4 LT, Neutrase 0.8 BrewQ and Flavourzyme showed no keratinolytic activity under the experimental conditions tested (Table 2). The absence of activity towards keratin azure by these proteases may be related to their substrate specificity being unsuitable for the keratin sequence. Keratinase activity of all enzymes was assayed at pH 8 and 10. Only Ronozyme ProAct showed improved activity at pH 10 (Table 2). Keratin absorption experiments were performed with Cibenza DP100 and Ronozyme ProAct to test the substrate binding capabilities of these enzymes. After incubation with hair, unbound soluble enzymes were assayed for protease activity with the azocasein assay. Cibenza DP100 retained 72% of activity, while Ronozyme ProAct retained 51% of caseinolytic activity (Table 2). The higher hair absorption capability of Ronozyme ProAct may be an important factor contributing to its superior keratinase activity.

**Table 2:** Protease and keratinase activities of commercial enzymes.

Enzyme	Substrate	pH	Specific Activity (10^3^ CU or KU/g of protein)
Cibenza DP100	Azocasein	8	10801 ± 788
	Azocasein (after hair absorption)	8	7778 ± 118
	Keratin azure	8	16 ± 1
		10	13 ± 1
Ronozyme ProAct	Azocasein	8	120127 ± 12578
	Azocasein (after hair absorption)	8	61324 ± 1561
	Keratin azure	8	178 ± 13
		10	262 ± 5
Multifect PR 6L	Azocasein	8	11 ± 1
	Keratin azure	8	0.13 ± 0.02
		10	0.33 ± 0.06
Alcalase 2.4 LT	Azocasein	8	11599 ± 2530
	Keratin azure	8	0 ± 0
		10	0 ± 0
Neutrase 0.8 BrewQ	Azocasein	8	7094 ± 571
	Keratin azure	8	0 ± 0
		10	0 ± 0
Flavourzyme	Azocasein	8	3949 ± 508
	Keratin azure	8	0 ± 0
		10	0 ± 0

One casein unit (CU) is defined as an increase of 0.1 absorption units at 440 nm after incubation with azocasein for 30 min at 37°C. One keratinase unit (KU) is defined as an increase of 0.1 in absorption units at 595 nm after incubation with keratin azure for 1 hour at 37°C. Data are represented as mean values ± standard deviation (n = 3).

### Mechanical properties of enzymatically treated hair

Multifect PR 6L, Cibenza DP100 and Ronozyme ProAct, the enzymes shown to have keratinolytic activity (Table 2), were applied to hair samples from cattle hides. Hairs were incubated with 0.2, 1 or 2 KU/mL Multifect PR 6L or Cibenza DP100, or 0.02, 0.1 or 0.2 KU/mL of Ronozyme ProAct for 16 h.

**Fig 1:**
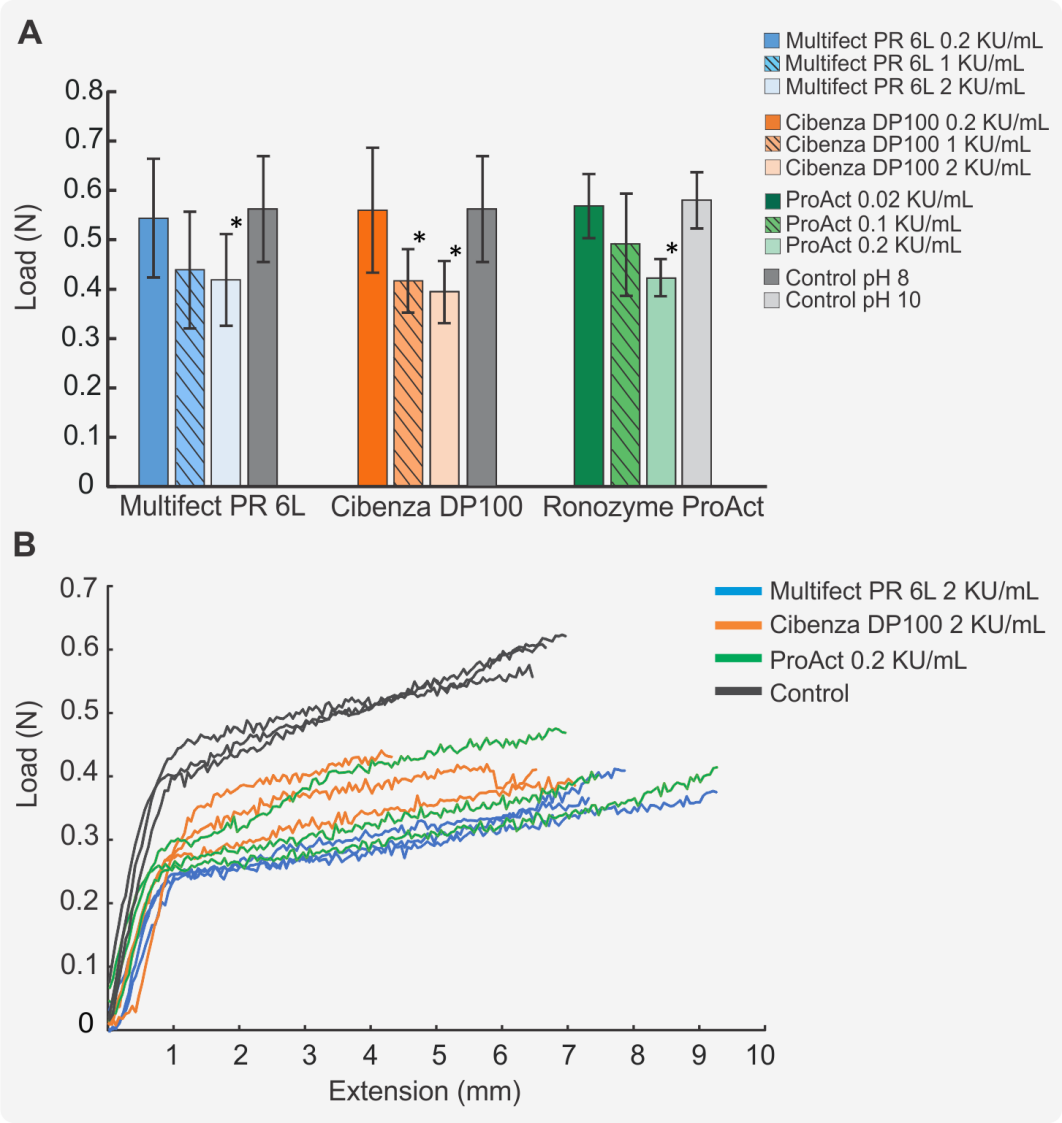
Load-extension curves of enzymatically treated hair. (A) Hair treated with 0.2, 1 or 2 KU/mL of Multifect PR 6 L or Cibenza DP100 at pH 8, or 0.02, 0.1 and 0.2 KU/mL of Ronozyme ProAct at pH 10. The control is defined as hair samples treated in buffer solution without enzyme. Values represent the load in Newtons required for hair to break. Data is represented as means ± SD (n = 10). *Significant statistical difference with control sample (p ≤ 0.01). (B) Load vs extension curves for the control, 2 KU/mL Multifect PR 6L, 2 KU/mL Cibenza DP100 and 0.2 KU/mL Ronozyme ProAct treated hair samples. The end point of each curve represents the force at the point of hair breakage.

Hair samples were clamped on a MTS Microforce Tryton 250 force meter and axial force testing was conducted at room temperature. Samples treated with 1 or 2 KU/mL Multifect PR 6L or Cibenza DP100 showed decreased breakage force compared to the untreated control, while treatment with 0.2 KU/mL of enzyme did not show any effect when compared to the control sample (Fig 1A and 1B). Ronozyme ProAct showed a greater effect on the hair fibre strength than the other enzymes despite the same number of enzyme units being present. An enzyme concentration as low as 0.2 KU/mL showed a marked decrease in the required hair breakage force (Fig 1A). Experiments could not be conducted at a higher concentration of Ronozyme ProAct because the shortening of the hair fibres due to degradation impaired clamping on the force meter.

Fig 1B shows load-extension curves for the no-enzyme control and enzymatically treated hair. A typical load-extension curve of a hair fibre shows three regions. A first linear section, the Hookean region, where the hair behaves mostly elastically, a transformation region where the α-helix coils uncoil and may transform into β- sheets, and a post-transformation region where the remaining α -helices and/or β- sheets are stretched until the hair reaches the breaking point [34, 35]. Curves in Fig 1B do not show an obvious transition point between the transformation and post-transformation regions. The lack of an obvious transition point is due to the low strain rate used in this study and might be consequence of a continuous α-β transformation before rupture that is commonly observed at low strain rates [36].

The hair samples treated with Cibenza DP 100, Multifect PR 6L or Ronozyme ProAct had a decreased resistance to stretch. The decreased resistance was revealed by the altered Hookean region of the load-extension curves of the enzymatically treated hair compared to the control samples. As a result, treated hair transitions from the Hookean region to the transformation region at a lower load (Fig 1A and 1B). The decrease in mechanical strength is most probably a result of enzymatic damage to the cortex. According to experiments performed by Robbins and Crawford, severe damage to the cuticle cannot be detected by tensile property evaluation. Only changes to the cortex can be detected by load-extension curves [37, 38].

### Scanning electron microscopy studies of enzymatic hair degradation

**Fig 2:**
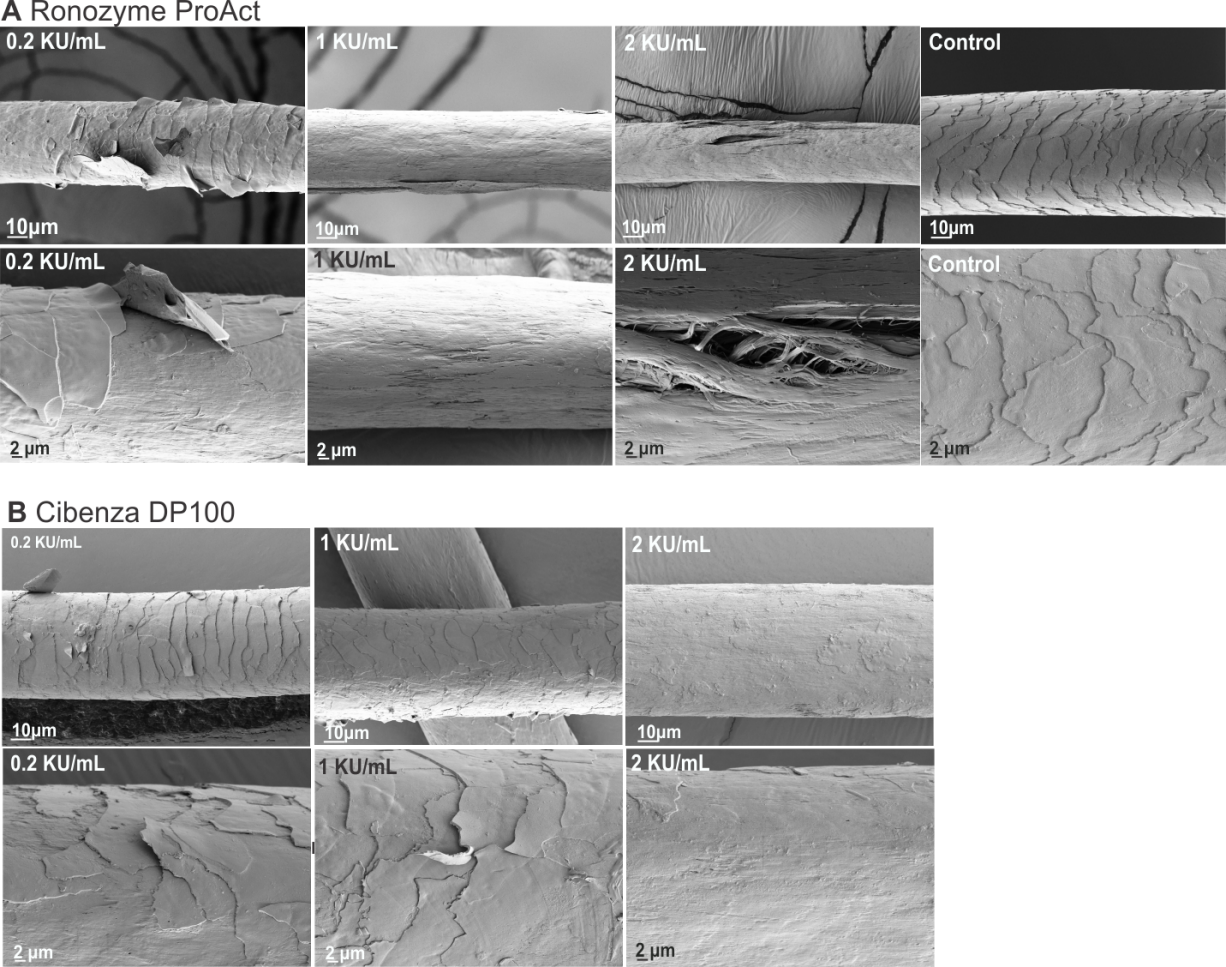
Degradation of hair samples by Cibenza DP100 and Ronozyme ProAct. Hair treated for 16 h with 0.2, 1 or 2 KU/mL of Ronozyme ProAct (A) or Cibenza DP100 (B). Control samples were incubated in buffer with no addition of enzyme. Two different images and magnifications (higher magnification on the bottom row of each set) are shown for each treatment.

Hair samples treated with 0.2, 1 or 2 KU/mL of Cibenza DP100 or Ronozyme ProAct for 16 h at 37°C were analysed by scanning electron microscopy. Hair degradation studies were not continued with Multifect PR 6L since this enzyme showed low keratinase activity when compared to Cibenza DP100 and Ronozyme ProAct (Table 2). Fig 2 shows representative SEM images obtained after each treatment. As expected, the increase in concentration from 0.2 to 2 KU/mL of enzyme increased damage to the hair fibre (Fig 2). While 0.2 KU/mL of Ronozyme ProAct showed lifting of the cuticle of the hair and most likely initial damage to the cortex as evidenced by the decrease in mechanical strength with this enzyme load (Figs 1 and 2), 1 KU/mL of this enzyme completely removed the cuticle and affected the cortex (Fig 2A). Treatment with 2 KU/mL of Ronozyme ProAct showed regions with extensive fractures to the hair fibres (Fig 2A). Treatment with Cibenza DP100 also showed increased damage with increasing concentration of enzyme, however, the extent of degradation of the hair fibre was reduced when compared to Ronozyme ProAct (Fig 2B). The cuticle was completely removed with 2 KU/mL of Cibenza DP100 and initial damage to the cortex was observed which correlates with the decreased force to break observed (Fig 1). Fig 2A and 2B shows control hair samples incubated in buffer with no addition of enzymes. In these samples, the cuticle cell surface exhibits entire cuticles, in a good general condition.

### Enzymatic degradation of hair and effect of reducing agents

Hair samples were treated for 16 h with Cibenza DP100 and Ronozyme ProAct with the addition of different concentrations of the reducing agents. The keratinolytic activity of Cibenza DP100 and Ronozyme ProAct in the presence of reducing agents was determined by the keratin azure assay (Table 3). Both enzymes showed increased activity in the presence of 1% sodium sulfite or 2% sodium thioglycolate. 2% Sodium sulfite or 2% or 5% sodium thioglycolate did not further improve nor inhibit the activity of Cibenza DP100, however, Ronozyme ProAct activity decreased in the presence of 2% sodium sulfite or 5% thioglycolate (Table 3). One keratinase unit (KU) is defined as an increase of 0.1 in absorption units at 595 nm after incubation with keratin azure for 1 hour at 37°C.

**Table 3:** Keratinase activity in the presence of reducing agents.

Enzyme	Reducing agent	pH	Specific Activity (10^3^ KU/g of protein)	Fold change in activity
**Cibenza DP100**	No reducing agent	8	16 ± 1	-
	1% sulfite	8	18 ± 1	1.1
	2% sulfite	8	18 ± 1	1.1
	1% thioglycolate	8	22 ± 2	1.4
	2% thioglycolate	8	23 ± 2	1.4
	5% thioglycolate	8	22 ± 2	1.4
**Ronozyme ProAct**	No reducing agent	10	262 ± 5	-
	1% sulfite	10	338 ± 2	1.3
	2% sulfite	10	235 ± 3	0.9
	1% thioglycolate	10	470 ± 14	1.8
	2% thioglycolate	10	684 ± 60	2.6
	5% thioglycolate	10	391 ± 18	1.5

Data are represented as mean values ± standard deviation (n = 3).

**Fig 3:**
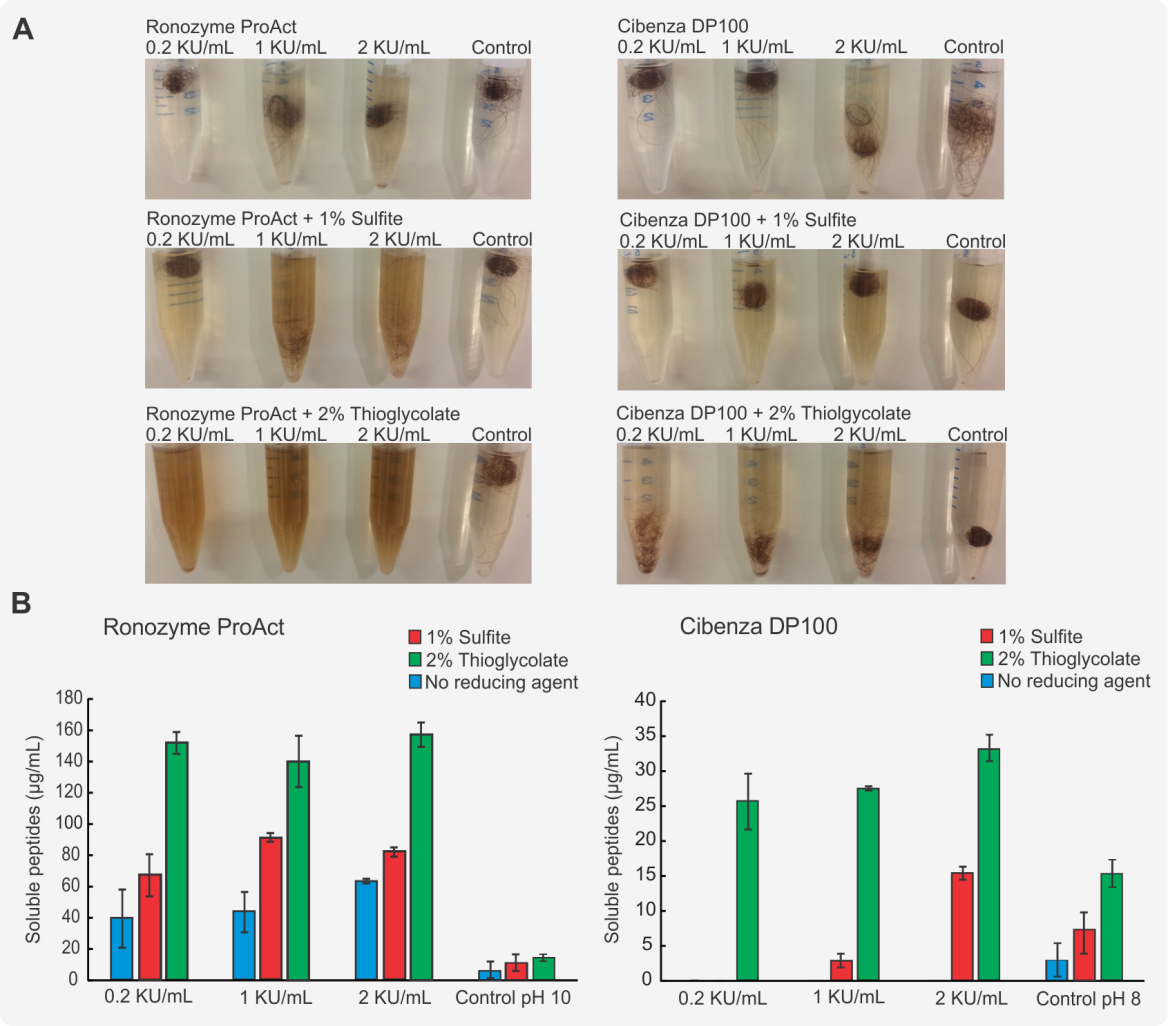
Enzymatic treatment of hair with or without reducing agents. (A) Hair samples treated with 0.2, 1 or 2 KU/mL Ronozyme ProAct or Cibenza DP100 for 16 h at 37°C with or without reducing agents. The extent of hair degradation can be seen by the disappearance of the hair fibre and the increased colouration of the solution, possibly due to melanin release from the brown hair. (B) Soluble peptides after hair treatment with 0.2, 1 or 2 KU/mL of Cibenza DP100 or Ronozyme ProAct for 16 h at 37°C in the presence of reducing agents. The control is defined as the hair sample treated in buffer solution without enzyme. Data are represented as means ± SD (n = 3).

The extent of hair degradation after enzymatic treatment in the presence of reducing agents was investigated by the measurement of released soluble peptides using the Bradford assay (Fig 3A and 3B). Since this colorimetric assay cannot detect free amino acids or peptides smaller than 3 kDa, the results of this study were interpreted as an approximation for comparative analysis between the degrading capabilities of the enzymes and the effect of the reducing agents and not as an absolute quantification of keratin breakdown.

Hairs were treated with 0.2, 1 or 2 KU/mL of Cibenza DP100 or Ronozyme ProAct with or without 1% of sodium sulfite or 2% sodium thioglycolate at 37°C. The degree of degradation of hairs into soluble peptides was markedly higher for Ronozyme ProAct than for Cibenza DP100 under the same conditions and the same loading of keratinase enzyme units (Fig 3A and 3B). The presence of reducing agent alone had some effect on hair degradation, which was observed by an increase in the concentration of soluble peptides in the no-enzyme control treated samples. The addition of reducing agents improved the effect of Cibenza DP100 and Ronozyme ProAct on hair degradation. From the two reducing agents tested, 2% sodium thioglycolate was the most beneficial for both enzymes (Fig 3B). In fact, Cibenza DP100 did not show detectable levels of soluble peptides unless reducing agent was added to the reaction.

Table 4 shows the percentage degradation of keratin for each treatment calculated by weight loss from the hair with the presence of reducing agent improving degradation. Sodium thioglycolate showed higher percentage degradation than sodium sulfite for all the enzyme concentrations tested, correlating with the results obtained by soluble peptide determination and the fold increase in keratinase activity of both enzymes (Table 3). Hair degradation exceeds 90% when treated with Ronozyme ProAct in the presence of sodium thioglycolate.

**Table 4:** Percentage keratin degradation.

Enzyme	Condition	Substrate	KU/mL	Average %
**Ronozyme ProAct**	Non reducing	Hair	0.2	24 ± 8
		Hair	1	35 ± 1
		Hair	2	38 ± 4
		Feather	2	23 ± 11
	1% sulfite	Hair	0.2	31 ± 4
		Hair	1	75 ± 8
		Hair	2	83 ± 4
		Feather	2	75 ± 4
	2% thioglycolate	Hair	0.2	92 ± 2
		Hair	1	91 ± 1
		Hair	2	95 ± 4
		Feather	2	97 ± 3
**Control no enzyme, pH 10**	Non reducing	Hair	-	0 ± 0
		Feather	-	0 ± 0
	1% sulfite	Hair	-	7 ± 5
		Feather	-	3 ± 3
	2% thioglycolate	Hair	-	7 ± 5
		Feather	-	7 ± 2
**Cibenza DP100**	Non reducing	Hair	0.2	15 ± 8
		Hair	1	18 ± 3
		Hair	2	34 ± 1
	1% sulfite	Hair	0.2	30 ± 6
		Hair	1	32 ± 4
		Hair	2	21 ± 8
	2% thioglycolate	Hair	0.2	43 ± 11
		Hair	1	49 ± 16
		Hair	2	59 ± 5
**Control no enzyme, pH 8**	Non reducing	Hair	-	0 ± 0
	1% sulfite	Hair	-	5 ± 7
	2% thioglycolate	Hair	-	8 ± 4

### Scanning electron microscopy studies of enzymatic hair degradation in the presence of reducing agents

**Fig 4:**
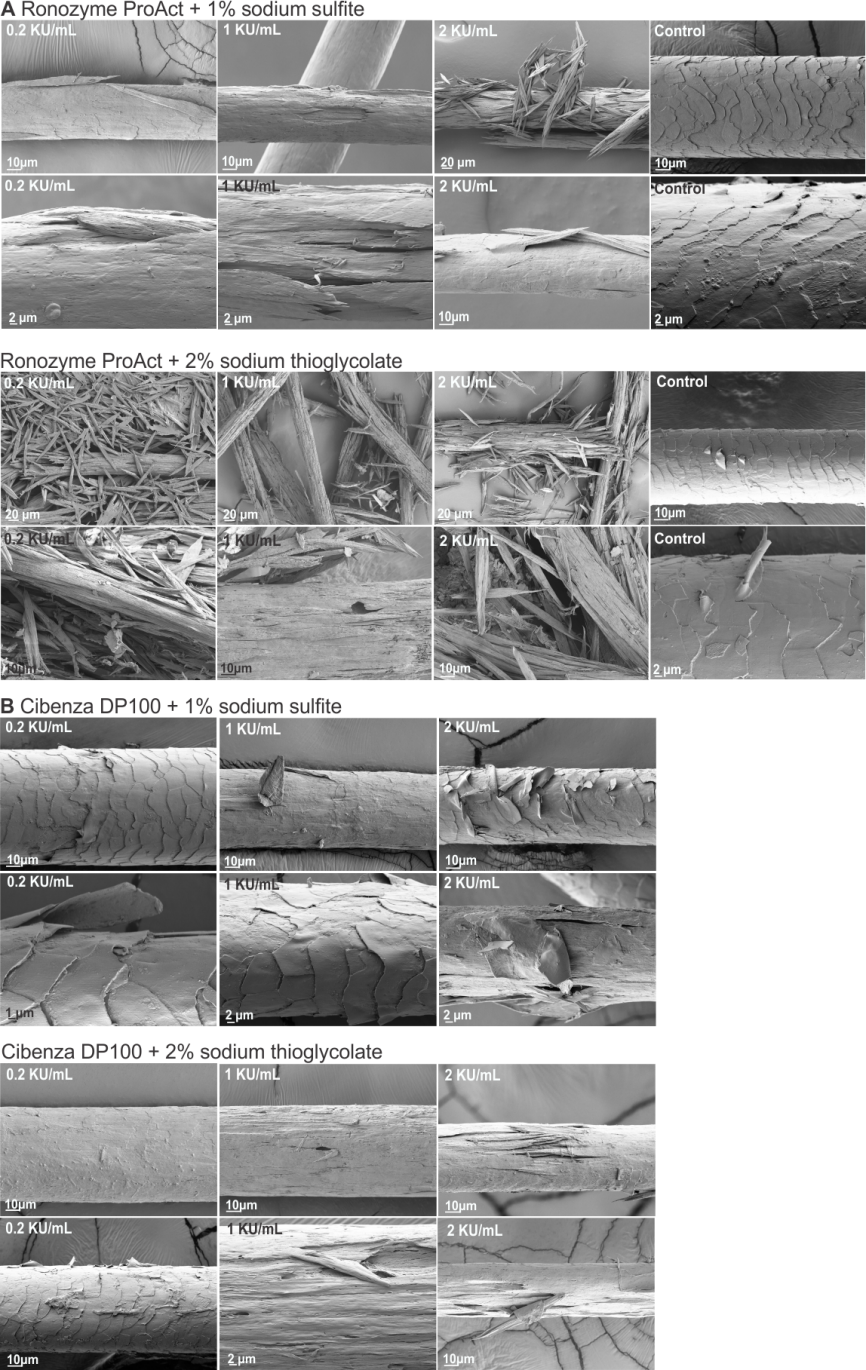
Degradation of hair samples by Ronozyme ProAct and Cibenza DP100 in the presence of reducing agents. Hair treated for 16 h with 0.2, 1 or 2 KU/mL of Ronozyme ProAct (A) or Cibenza DP100 (B) with 1% sodium sulfite or with 2% sodium thioglycolate. Control samples treated without enzyme, with or without reducing agents. Two different images and magnifications (higher magnification on the bottom row of each set) are shown for each treatment.

Hair samples treated with 0.2, 1 or 2 KU/mL of Cibenza DP100 or Ronozyme ProAct in the presence of reducing agent for 16 h at 37°C were analysed by scanning electron microscopy. Fig 4 shows representative SEM images obtained after each treatment.

The addition of reducing agents to enzymatic treatments had a clear effect on hair degradation for both enzymes, correlating with improved enzymatic activity and increased percentage degradation (Tables 3 and 4). The presence of 1% sodium sulfite in hair treatments with 0.2 KU/mL Ronozyme ProAct showed a complete removal of the cuticle surface and initial damage to the cortex (Fig 4A). Extensive fracturing of the hair fibre was observed with 1 KU/mL or 2 KU/mL of Ronozyme ProAct under this condition (Fig 4A). The addition of 1% sodium sulfite to enzymatic treatments with Cibenza DP100 enhanced hair degradation; extensive removal of the hair cuticle was observed with 1 KU/mL, while hair fractures could be detected with 2 KU/mL of enzyme (Fig 4B). Control samples treated with sodium sulfite without enzyme showed minor lifting of the cuticle surface in same areas (Fig 4A and 4B).

Enzymatic hair treatments conducted in the presence of 2% sodium thioglycolate revealed extended hair structure degradation. The effect was more obvious for Ronozyme ProAct than Cibenza DP100 (Fig 4). 0.2, 1 or 2 KU/mL of Ronozyme ProAct completely degraded the hair fibre in presence of 2% sodium thioglycolate (Fig 4A), correlating with 92, 91 and 95% degradation for 0.2, 1 or 2 KU/mL of Ronozyme ProAct (Table 4). The fracturing was very significant, only small fragments of hair could be observed during scanning electron microscopy analysis. Addition of sodium thioglycolate to Cibenza DP100 treatments improved keratinase activity (Tables 3 and 4 and Fig 4B). Hair fracturing was evident with 2 KU/mL of enzyme under this condition, however, the damage to the hair fibre was reduced when compared with Ronozyme ProAct treated samples. Percentage of degradation with sodium thioglycolate was 43, 49 and 59% for 0.2, 1 or 2 KU/mL of Cibenza DP100 (Table 4). Control samples without enzyme showed that the presence of sodium thioglycolate caused some lifting of the hair cuticle (Fig 4A and 4B).

### Time dependent enzymatic keratin degradation

**Fig 5:**
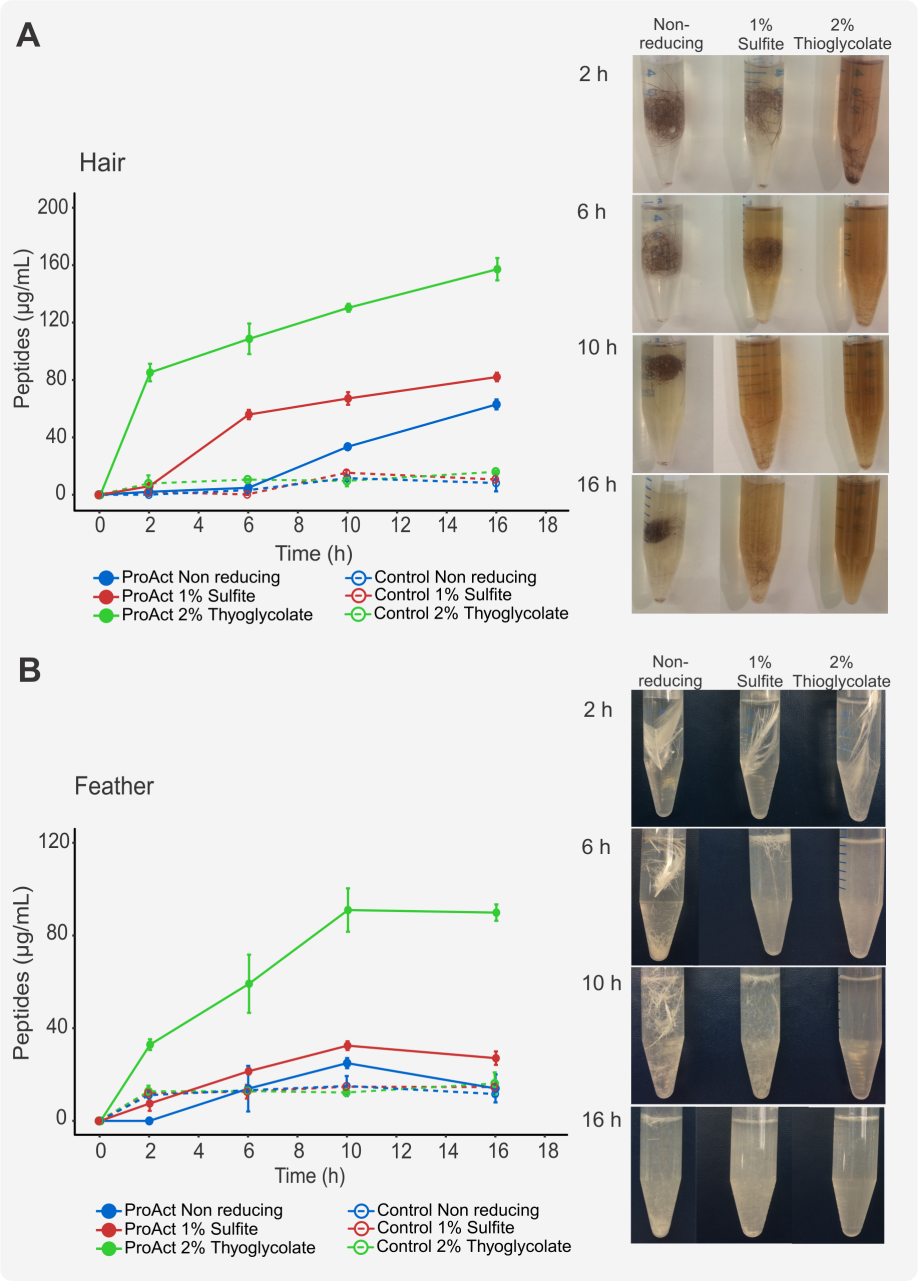
Time series studies of degradation of hair and feathers. Soluble peptides (μg/mL) after hair (A) and feather (B) treatment with 2 KU/mL of Ronozyme ProAct with or without reducing agents at 37°C. The control is defined as hair or feather sample treated in buffer solution without enzyme. Data is represented as means ± SD (n = 2).

Hair and feather samples were both included in the following studies to investigate the dynamics of keratin enzymatic breakdown for two different substrates. Both hair and feathers were treated with 2 KU/mL of Ronozyme ProAct with or without reducing agents and the resulting soluble peptide concentration was quantified using the Bradford assay at different time points (2, 6, 10 and 16 h) (Fig 5). Since Ronozyme ProAct showed better keratinolytic activity compared to Cibenza DP100, these studies were performed with this enzyme only.

Correlating with previous results, the degree of degradation of hair and feathers was clearly enhanced by the addition of reducing agent. The presence of 2% thioglycolate decreased the time for enzymatic degradation, the most extensive decomposition occurred during the first two hours of treatment under this condition. Furthermore, both hair and feather samples were extensively degraded after 6 h treatment, as observed in the photographs in Fig 5. The same effect, although not as marked, was observed for 1% sulfite.

**Fig 6:**
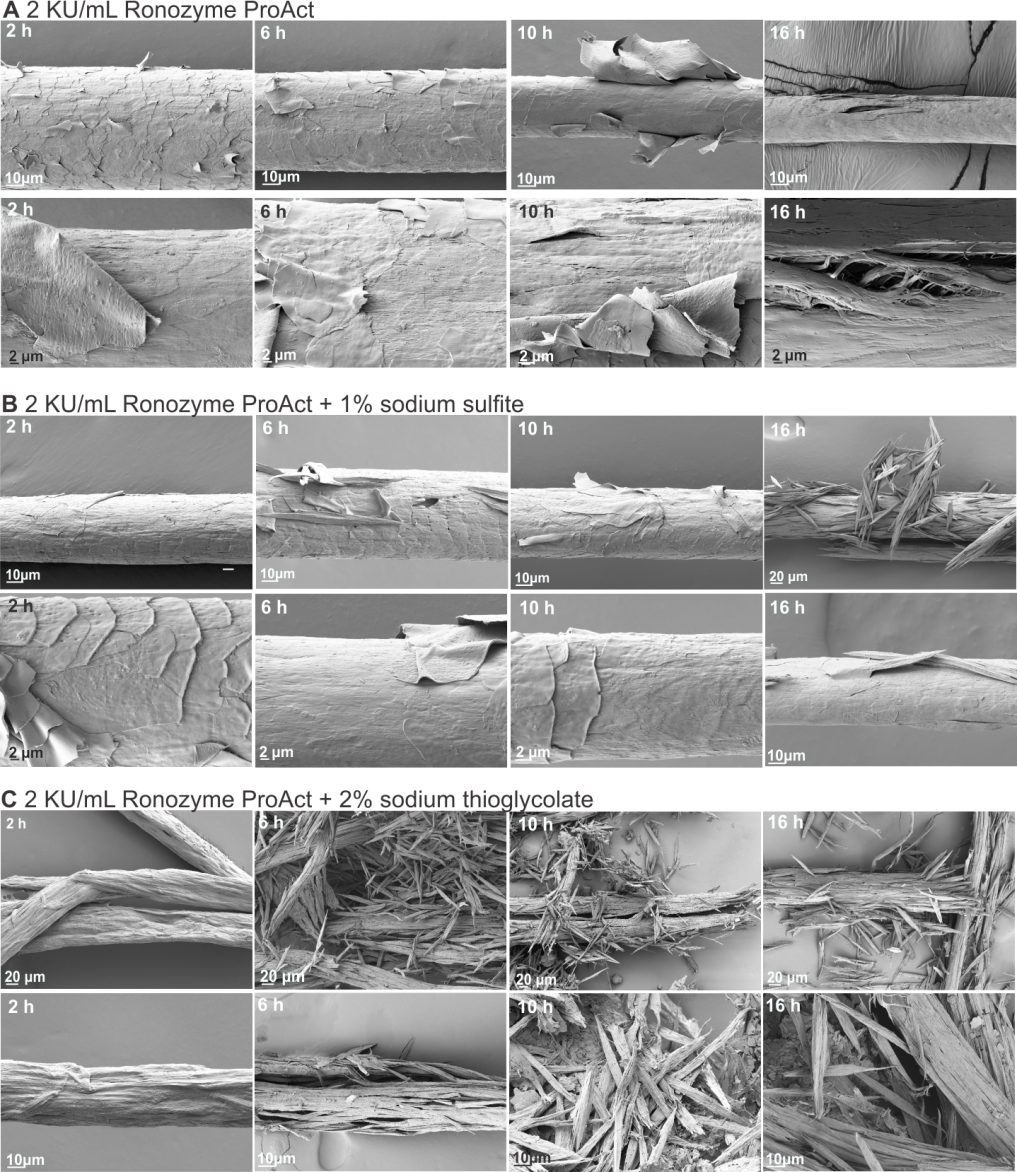
Scanning electron microscopy of the progression of hair enzymatic degradation. Hair treated with 2 KU/mL Ronozyme ProAct without reducing agent (A), with 1% sodium sulfite (B) or with 2% sodium thioglycolate (C) for 2, 6, 10 and 16 h at 37ᵒC. Two different images and magnifications (higher magnification in the lower row of each set) are shown for each time and treatment.

Hair and feather samples from the different time points of treatment with Ronozyme ProAct (2, 6, 10 and 16 h) were analysed by scanning electron microscopy. Well-defined stages of hair and feather decomposition were identified. In the case of hair, the first stage of degradation was lifting of the cuticle until complete depletion. This process occurred from 0 to 6 h of treatment with 2 KU/mL of Ronozyme ProAct without reducing agent. From 6 h onwards, the cortex of the hair appeared affected, with small fracturing detectable, followed by more extensive hair fibre fracturing at around 16 h of treatment (Fig 6A). The addition of reducing agent facilitated the degradation process, as previously shown (Fig 4). For 1% sodium sulfite, variability in the degree of degradation was observed for samples taken at 6 and 10 h of treatment with Ronozyme ProAct. Some samples suffered cortex fracturing as soon as 6 h while other hairs retained part of the cuticle until 10 h of treatment (Fig 6B). Complete depletion of the cuticle layer was observed at 16 h of treatment under this condition, with extensive fracturing of the hair fibre compared with enzymatic treatment without reducing agent (Fig 6A). Two hours of keratinase treatment in the presence of 2% sodium thioglycolate showed significant damage to the hair fibre and no cuticle remaining. Notably, samples were splintered after 6 h of treatment under this condition (Fig 6C). This treatment extensively degraded the hair fibre in a shorter period of time than any of the other treatments.

**Fig 7:**
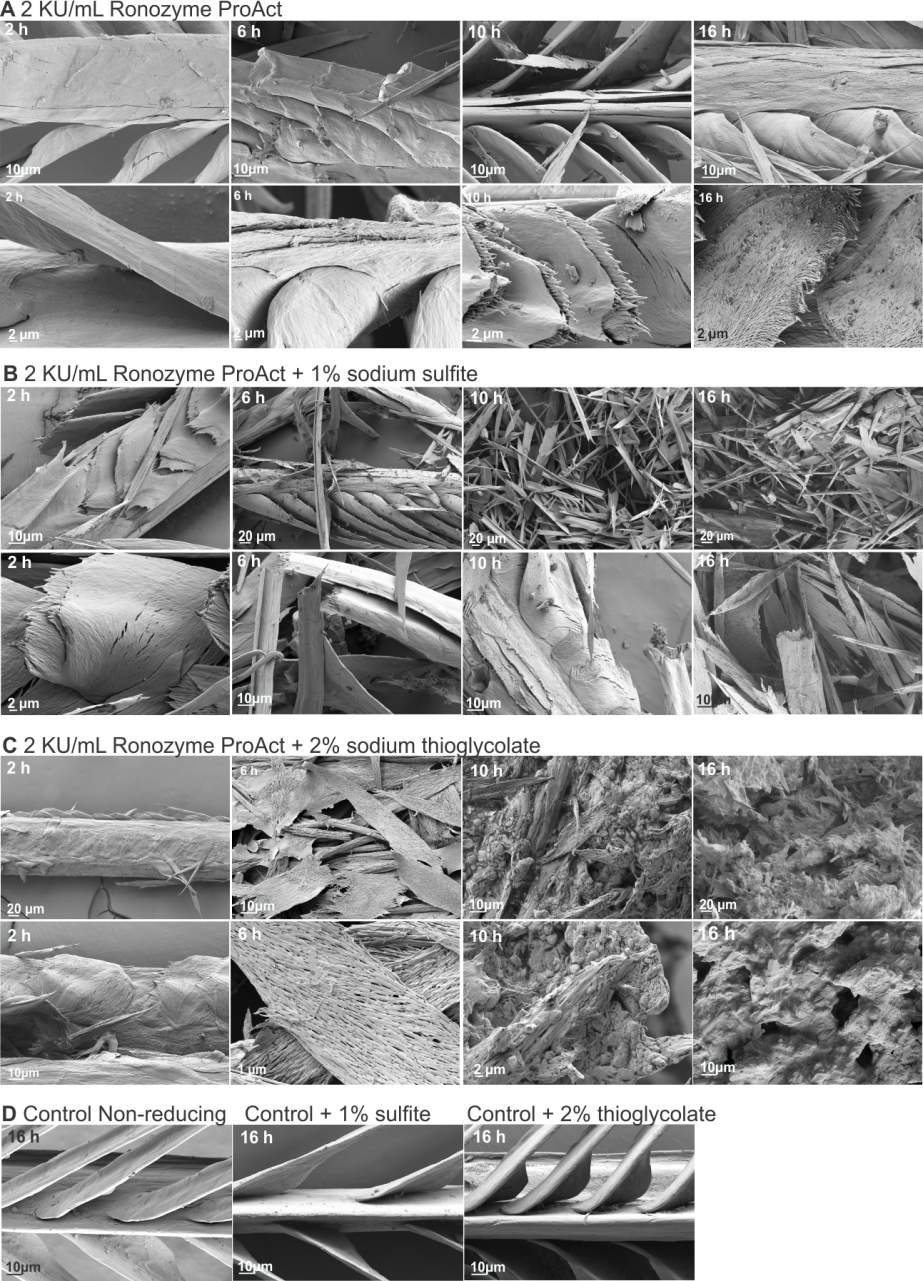
Scanning electron microscopy of the progression of feather enzymatic degradation. Feathers treated with 2 KU/mL Ronozyme ProAct without reducing agent (A), with 1% sodium sulfite (B) or with 2% sodium thioglycolate (C) for 2, 6, 10 and 16 h at 37°C. Two different images and magnifications (higher magnification in the lower row of each set) are shown for each time and treatment.

Keratin degradation from feathers also revealed well-defined stages of decomposition (Fig 7). After 2 h treatment with 2 KU/mL of Ronozyme ProAct some fractures were revealed on barbs, however, extensive fracturing and complete breaking of barbules was observed from 6 h of treatment onwards (Fig 7A). After 16 h, the structure of the feather was transformed in a material with porous appearance. The addition of reducing agents to the treatment decreased the time required for the enzymatic degradation process (Fig 7B and 7C), as observed for hair samples. Marked fracturing of barbs and barbules were observed after 2 h treatment with Ronozyme ProAct in the presence of 1% sodium sulfite and a great extent of degradation after 6 h of treatment (Fig 7B). The addition of 2% sodium thioglycolate to the enzymatic treatment had a strong effect on feather structure, after 2 h incubation feathers were degraded into a porous keratin material (Fig 7C). At 6 h of treatment, feathers were transformed into sheets of porous keratin and from 10 h onwards the structure was completely degraded to an amorphous protein material (Fig 7C).

## Discussion

We used multiple analyses to investigate the degradation of hair and feather keratin by enzymes. We tested several commercial proteases for keratinolytic activity and the ones that showed activity towards keratin were used in mechanical, biochemical and microscopic studies to characterize the enzymatic process of keratin breakdown.

Hair mechanical strength studies were carried out to investigate the weakening of the hair fibre after enzymatic treatment. The analysis of load versus extension curves showed that treatment with the enzymes decreased the hair breakage force. Scanning electron microscopy showed that the weakening of the hair fibre causing premature rupture was related to the loss of cuticle and the weakening of the hair cortex [37].

We also investigated the effect of reducing agents on the enzymatic degradation. Keratin is very recalcitrant due to its high degree of disulfide bonds. Initial attack of the disulfide bonds by reducing agents facilitates proteolytic activity by enzymes. We tested two reducing agents, sodium sulfite and sodium thioglycolate. Both compounds markedly improved enzymatic degradation of keratin, however, sodium thioglycolate was more effective than sodium sulfite, as evidenced by soluble peptide quantification, percentage degradation and scanning electron microscopy. Sodium thioglycolate may be more effective due to a higher reducing potential towards disulfide bonds compared to sodium sulfite. However, no experiments were conducted to confirm this hypothesis.

For the two proteases that showed keratinase activity, Cibenza DP100 and Ronozyme ProAct, we observed that Ronozyme ProAct was the best enzyme for keratin degradation. Cibenza DP100 is composed of *B*. *licheniformis* PWD-1 fermentation solubles, containing KerA, a well-known subtilisin-type keratinase [33]. Ronozyme ProAct is a serine protease from *Nocardiopsis prasina* described as a chymotrypsin-type protease and not marketed as a keratinase. Some keratinolytic enzymes from *Nocardiopsis* have been described, one example is NapA from *Nocardiopsis spp*. TOA-1, also a chymotrypsin-type protease [39]. Saha et al also isolated a potent keratinolytic *Nocardiopsis* strain from poultry waste capable of completely degrading feathers [40].

Some authors suggest that all proteases belonging to the subtilisin family could be able to degrade keratin in the presence of reducing agent or disulfide reductase, and that microorganisms that fail to grow on keratin might lack disulfide reducing potential [41, 42]. Alcalase 2.4 LT, a subtilisin-type serine protease from *B*. *licheniformis* without keratinase activity, showed very low activity in the keratin azure assay in the presence of 1% sodium sulfite (0.30 ± 0.03 KU/g) and no activity when 2% sodium thyoglycolate was added to the reaction. According to this result, it might be possible that all subtilisin-type proteases have some activity against keratin substrates, yet, what defines a keratinase (or a superior keratinase) is that it does not rely on the presence of reducing agents or a disulfide reductase. Undoubtedly, in presence of the same amount of reducing agent and keratinase units, the enzyme from *Nocardiopis prasina* (Ronozyme ProAct) was much more effective in hair and feather degradation than *B*. *licheniformis* PWD-1 KerA enzyme (Cibenza DP100).

Substrate specificity of keratinases has only been partially described. Most keratinases seem to prefer hydrophobic and aromatic amino acids at position P1, including KerA from *B*. *licheniformis* PWD-1 and NapA from *Nocardiopsis spp*. TOA-1 [39, 43, 44]. The enzymatic cleavage of the peptide bonds of keratin is inherently difficult because of the restricted enzyme-substrate interaction on the surface of the keratin fibres. The hydrolysing ability of keratinolytic proteases may be related to their capacity to bind to compact substrates and a more exposed active site [45]. Similar to chitinases, where C-terminal domains enable interaction with the compact and insoluble chitin substrate, a binding domain could be part of the keratinase structure facilitating keratin binding [45, 46]. The C-terminal domain of Vpr serine protease from feather degrading *B*. *cereus* DCUW dictates the substrate specificity [47]. The elimination of this domain in recombinant variants of Vpr protease showed no ability to degrade feathers, yet maintained activity towards casein and gelatine [47]. Similar substrate specificity functions were demonstrated for the C-terminal domains of keratinases from *Stenotrophonomonas* spp. [48]. Keratin absorption experiments were performed with Cibenza DP100 and Ronozyme ProAct to test the hair binding capabilities of these enzymes. Cibenza DP100 retained 21% more protease activity than of Ronozyme ProAct. Improved absorption to the substrate might reflect superior keratinase activity of Ronozyme ProAct. Mitsuiki et al found that NAPase from *Nocardiopsis* sp. TOA-1 has a strong absorption capability towards keratin, possibly due to the presence of an efficient binding pocket for keratin [39].

Keratin breakdown was also investigated by scanning electron microscopy at different time points of enzymatic treatment. We observed well-defined stages of keratin degradation. In the case of hair, the lifting of the hair cuticle was the first step of degradation, followed by its complete removal and initial damage of the cortex. The next stage of decomposition was fracturing of the cortex that, in presence of reducing agents, was extended to the generation of small fragments of keratin that were finally converted to an amorphous protein material. In the case of feathers, initial fracturing of the barbs was observed followed by extensive rupture of barbs and barbules. After this, the remaining structure of the feather was converted in a material with porous appearance and, finally, in the presence of reducing agents, into amorphous protein material.

Most research on keratinases has been done with keratinolytic microorganisms using the whole cell culture broth to test for keratin decomposition capabilities. The time frame usually observed in these investigations for the complete breakdown of keratin substrates varies from one to several days [18, 42, 49–53]. Some industrial applications may require a shorter application time. Several enzymatic formulations are still based on whole cell systems but the use of isolated enzymes presents many advantages, including faster scale up, avoidance of unwanted side reactions, and easier storage and transport. There are only a limited number of reports showing the effective degradation of keratin using purified or recombinant keratinases, where percentage degradation is variable and time frames fall into the 24–72 h range [45, 54–56]. We report here a keratinase with the ability to almost completely degrade keratin (exceeding 90%) in 16 h, a period of time adequate for industrial applications. The degradation experiments in this work where conducted at 37°C, and Ronozyme ProAct is stable and has improved activity towards keratin azure at 50°C (584 ± 182 10^3^ KU/g), hence the breakdown process could be further reduced in time if needed for a particular application.

**Fig 8:**
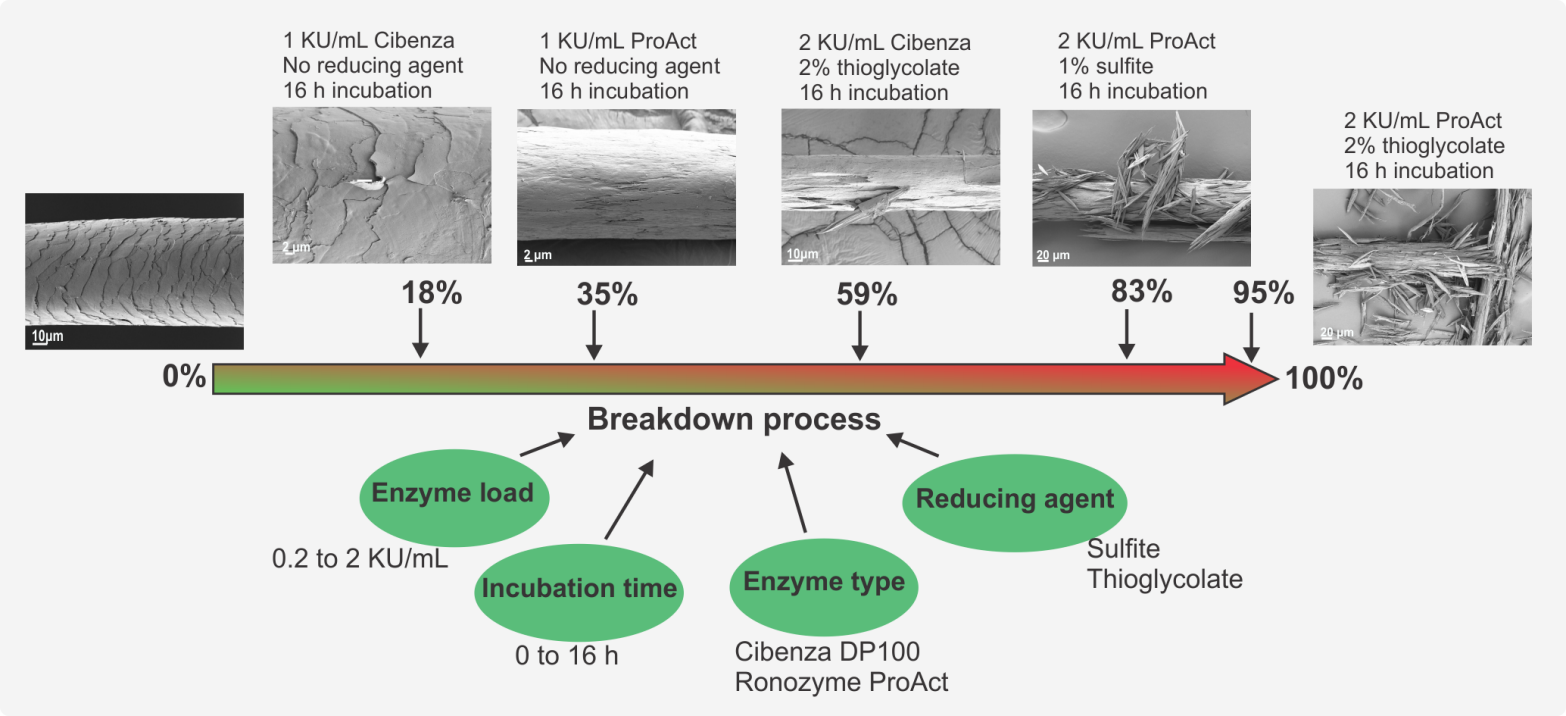
Keratin weakening and hydrolysis. Weakening and hydrolysis of keratin can be optimised for a particular application. Figure shows the conditions tested in this work and some of the results obtained.

The poultry, slaughterhouse and leather processing industries generate considerable amount of keratin waste that is commonly processed by thermal or thermo-chemical treatment [26]. Soluble peptides obtained from the enzymatic degradation of keratin waste could be incorporated in animal feed or used as nitrogen source in fermentations to produce other value bio-products. These soluble peptides have more nutritional value than those obtained through thermal or thermo-chemical hydrolysis [27]. Some other applications, for example in the cosmetic or pharmaceutical industries, might require only weakening of the keratin fibre and not complete degradation. Time series analysis of keratin breakdown is essential to determine appropriate application times and/or enzyme load. We have shown that such important aspects can be controlled and optimized (Fig 8).

The results presented in this work provide new insights into the keratin degradation process by enzymes. We show that removal of the hair protective cuticle and damage to the cortex affects mechanical strength and is the initial step of enzymatic attack. We demonstrate that the presence of reducing agent is essential for complete degradation, with thiogycolate providing improved enzymatic activity allowing faster treatment periods than previously reported. Finally, we proved that the enzymatic treatment used in this work can be further applied to other keratin substrates like feathers, forming a basis for improved application of keratinases in a variety of industries.
